# Local Factors Rather than the Landscape Context Explain Species Richness and Functional Trait Diversity and Responses of Plant Assemblages of Mediterranean Cereal Field Margins

**DOI:** 10.3390/plants9060778

**Published:** 2020-06-22

**Authors:** Yesica Pallavicini, Fernando Bastida, Eva Hernández-Plaza, Sandrine Petit, Jordi Izquierdo, Jose L. Gonzalez-Andujar

**Affiliations:** 1 Instituto de Agricultura Sostenible (CSIC), Avda. Menendez Pidal s/n, Aptdo 4084, 14080 Córdoba, Spain; yesipalla@gmail.com (Y.P.); mehernan@ias.csic.es (E.H.-P.); 2 Departamento de Ciencias Agroforestales, Universidad de Huelva, Campus La Rábida, Ctra. Palos s/n, Palos de la Frontera, 21819 Huelva, Spain; bastida@uhu.es; 3 Institut National de la Recherche Agronomique (INRA), UMR 1347 Agroécologie, 17 rue Sully, BP 86510, F-21065 Dijon CEDEX, France; sandrine.petit-michaut@inrae.fr; 4 Departament d’Enginyeria Agroalimentària i Biotecnologia, Universitat Politècnica de Catalunya, C/Esteve Terradas 8, Castelldefels, 08860 Barcelona, Spain; jordi.izquierdo@upc.edu

**Keywords:** plant diversity, plant functional traits, agricultural intensification, seed mass

## Abstract

Arable field margins are valuable habitats providing a wide range of ecosystem services in rural landscapes. Agricultural intensification in recent decades has been a major cause of decline in plant diversity in these habitats. However, the concomitant effects on plant functional diversity are less documented, particularly in Mediterranean areas. In this paper, we analyzed the effect of margin width and surrounding landscape (cover and diversity of land use and field size), used as proxies for management intensity at local and landscape scales, on plant species richness, functional diversity and functional trait values in margins of winter cereal fields in southern Spain. Five functional traits were selected: life form, growth form, seed mass, seed dispersal mode and pollination type. RLQ and fourth-corner analyses were used to link functional traits and landscape variables. A total of 306 plant species were recorded. Species richness and functional diversity were positively related to margin width but showed no response to landscape variables. Functional trait values were affected neither by the local nor landscape variables. Our results suggest that increasing the margin width of conventionally managed cereal fields would enhance both taxonomic and functional diversity of margin plant assemblages, and thus the services they provide to the agro-ecosystem.

## 1. Introduction

Agricultural intensification is a worldwide phenomenon [1,2] adversely impacting biodiversity and ecosystem services within agricultural systems [3,4]. The landmark features of agricultural intensification include increased crop management intensity, e.g., high use of agrochemicals and short rotation schemes [1,2,3] and decreased landscape complexity resulting from a higher proportion of land designated for annual crops at the expense of land-use diversity, semi-natural habitats and field margins [3,4,5,6,7].

Field margins can contribute to alleviating the negative effects of agricultural intensification on biodiversity through their ability to sustain populations of pollinators, natural enemies and plant species providing ecosystem services [8,9,10,11]. Additional services provided by field margins include reduced soil loss to erosion and protection of watercourses from pesticide and fertilizer drifts [12,13]. These habitats harbor considerably higher plant, vertebrate and invertebrate diversity than adjacent fields [14,15]. The significant role of field margins in agro-ecosystems has prompted efforts to investigate the impacts of agricultural intensification on margin plant communities [16]. Locally, the management of margins, as well as the farming system in place in the adjacent crop, can alter both the richness and the composition of plant communities in these habitats [16,17,18]. At broader spatial scales, the compositional diversity and structural complexity of the surrounding landscape may affect the plant composition of field margins, although the effects reported in the literature are inconsistent [15,16,17,18,19,20,21]. In addition, local and landscape factors affecting margin plant communities have mostly been described in temperate Northern and Central Europe [12,13], whereas in Mediterranean Europe, they have been poorly documented [16]. Apart from a contrasting climate, Mediterranean cereal areas also differ from their counterparts in temperate Europe in physiognomic features, particularly the occurrence of remarkably narrow field margins [10,22,23].

Agricultural intensification at local and landscape scales may also impact the functional diversity of plant communities regardless of the effects on taxonomic diversity and community composition [24]. This process can occur via the filtering out of plant species with functional traits maladapted to the new environmental conditions [25,26]. As a consequence, the initial distributions of trait values are shifted towards the most successful trait combination, with the resulting narrowing of trait values, and thus of functional diversity, potentially having detrimental effects on the provision of ecosystem services [27]. Such processes have been described in the flora of arable fields [28,29] suggesting that in some cases, species richness and functional responses can be decoupled [30], although, to date, it has not been documented in arable field margins.

The objective of this paper was to assess whether the intensity of agricultural management, measured at the local scale (as field margin width) and at the landscape scale (as cover of different land uses, land-use diversity and field size), affect species richness and functional traits of plant assemblages of margins of conventionally managed Mediterranean cereal fields. The objective is framed in the following hypotheses: (1) species richness and functional diversity increases with margin width and landscape complexity; (2) life form, growth form, pollination type, seed dispersal mode and seed mass are responsive to margin width and surrounding landscape complexity.

## 2. Results

A total of 306 plant species (Appendix A) were recorded, with an average richness of 21 ± 9 species per margin. The most frequent species were arable weeds typical of Mediterranean cereal cropping systems: *Lolium rigidum* Gaudin (73%), *Avena sterilis* L. (56%), *Hordeum murinum* L. (51%) and *Anagallis arvensis* L. (50%) (Appendix B). We detected only one species, *Malvella sherardiana* Jaub. and Spach, cataloged as endangered [31]. Fifty-eight species occurred in more than 10% of the sampled margins and were used for computing functional diversity indices and both three-table analyses. These species were mostly therophytes, forbs, entomogamous and barochorous (Table 1). There were also many wind-dispersed species including *Sonchus oleraceus* L., *Sylibum marianum* (L.) Gaertn., *Lactuca serriola* L. and *Papaver rhoeas* L., which are considered colonizers in arable cropping systems [32].

### 2.1. Landscape Complexity Gradient

**Figure 1:**
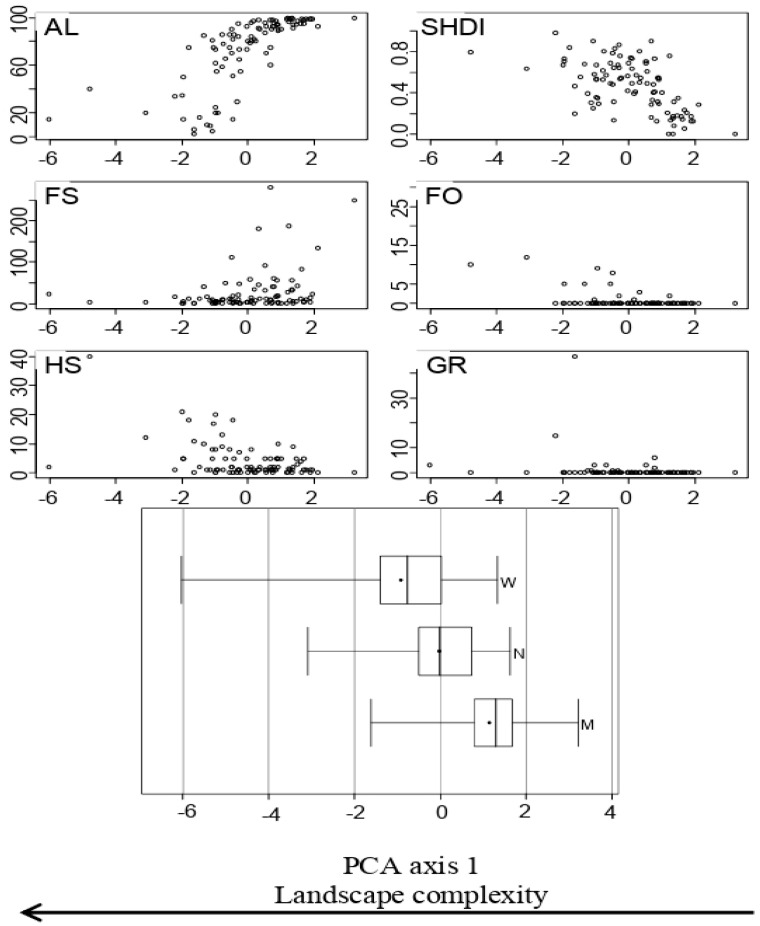
Landscape variables significantly associated with PCA axis 1. AL = arable land cover, SHDI = Shannon diversity index of land-use types, FS = field size, FO = woodland cover, GR = annual grassland cover, HS = cover of human settlements, and width = margin width class, N: narrow margins (<1 m), M: Medium-sized margins (1–2 m), W: wide margins (>2 m). Boxplots show median values (bold line) and mean (dot); box limits represent lower an upper quartiles, and whiskers represent the minimum and maximum values.

The first two axes of the PCA analysis accounted for 40% of the total variability (PC1, 24%; PC2, 16%). All the seven local and landscape variables showed a stronger correlation with PC1 than with PC2, and thus, only the first axis was further considered. PC1 represented a gradient of landscape complexity (Table 2, Figure 1). It splits margins from those embedded in more complex landscapes (low field size, high land-use diversity and high cover of grassland, woodland and human settlements) to those in simpler landscapes (large field size, high cover of arable land). The margin width type was partially associated with the gradient of landscape complexity (PC1, Table 2). Wide margins were more frequent in complex landscapes, while medium-sized margins were more frequent in simple landscapes. Narrow margins were evenly located in both complex and simple landscapes (Figure 1).

### 2.2. Agricultural Intensification Effects on Species Richness, Functional Diversity and Functional Traits

**Figure 2:**
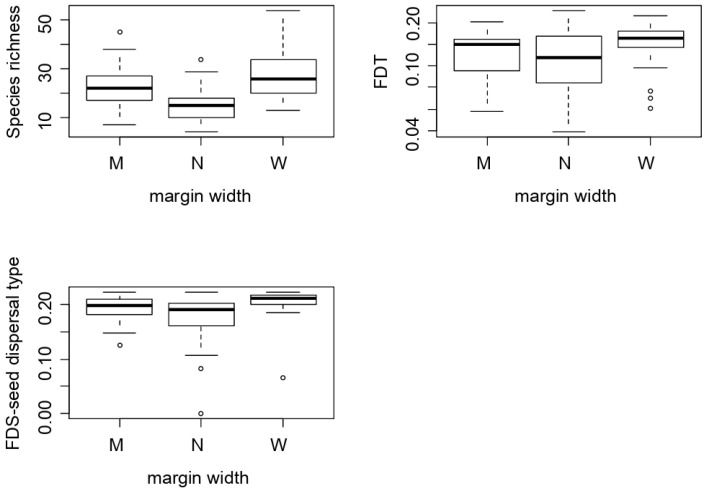
Relationship between species richness, functional diversity for the whole trait set (FDT) and for seed dispersal mode (FDS-seed dispersal), and margin width class (N, narrow margins, <1 m width; M, medium sized margins, 1–2 m width; W, wide margins, >2 m width). Boxplots show median values (bold line), box limits represent lower and upper quartiles and whiskers represent minimum and maximum values.

Species richness, functional diversity for the full trait set and for seed dispersal mode were affected by margin width (Table 3, Figure 2). Wide (28 ± 10 species) and medium-sized margins (23 ± 9 species) had significantly higher species’ richness than narrow margins (15 ± 6 species). Functional diversity, as measured for the full trait set, was significantly higher in wide margins than in narrow margins (Table 3, Figure 2), whereas for seed dispersal mode, it was significantly higher in wide margins than in medium and narrow margins (Table 3, Figure 2). Neither species richness nor any functional diversity variables were correlated with the landscape complexity gradient (PC1) or with any individual landscape variable (Table 3).

The RLQ analysis did not reveal any shift in functional trait values in response to local and landscape variables. Random permutations of the rows of the R and Q tables, used to test respectively whether species presence is independent or not from the environment and from trait values, showed that both null hypotheses could not be rejected (*p* = 0.27 and *p* = 0.30, respectively). This joint result indicated a lack of support for an association between intensification variables and functional trait values. In concordance with the RLQ analysis, the fourth-corner analysis detected no significant association between individual trait values and margin width or any individual landscape variable (Table 4).

## 3. Discussion

In this study, we investigated the role of margin width and landscape complexity on species richness, functional diversity, and functional trait values of plant assemblages of margins of conventionally managed winter cereal fields in Southern Spain. Our results suggested that margin width had a significant effect on species richness and functional diversity of margin plant communities, whereas the effects of the landscape context appeared to be of minor importance. In addition, we showed that life form, growth form, seed mass, seed dispersal mode and pollination type in these plant communities were responsive to neither margin width nor landscape complexity.

According to our expectations, a positive effect of margin width was detected both on plant species richness and functional diversity. Unsurprisingly, wider margins were found to support higher taxonomic diversity than narrow margins. Quantitatively, margins wider than 1 m (medium and wide) harbored 40% more species than narrower ones [16,17,18]. Apart from a direct effect of width resulting from the increased area, higher diversity can also be gained through increased habitat heterogeneity in wider margins [37]. In parallel, a number of studies have provided evidence that narrow margins are strongly impacted by disturbances associated with the agricultural management of the adjacent field (e.g., herbicide drift and nutrient leaching) while wider margins gain a buffering capacity in front of disturbances [17,37,38]. Our study also showed that wide margins (>2 m) harbored a higher plant functional diversity than narrow ones (<1 m), i.e., allowed the coexistence of plants exhibiting dissimilar functional trait values, as found for the full trait space and for seed dispersal mode. These results suggest that taxonomic and functional diversity were not decoupled here, i.e., that the loss of species translated into losses in functions. This finding reinforces the view that Mediterranean field margins of conventionally managed cereal crops often shelter a low functional diversity, most likely due to their narrow character, which renders them disturbance-prone, and to a restricted species pool in the surrounding, mostly simplified landscape [30]. Indeed, the most frequent therophytes recorded here are pernicious weeds of winter cereal crops [39], which would suggest that narrower margins experience environmental conditions similar to those of crop fields.

The taxonomic and functional plant responses were mostly driven here by agricultural intensification at the field scale (margin width), rather than at the landscape level. Although landscape-scale effects on field margin plant diversity have been described in some studies [15,16,28,40], our results are in line with other studies suggesting an overriding role of local management on arable plant taxonomic and functional diversity [19,21,30,41]. This lack of landscape effects may have several plausible causes that could act in isolation or in combination: (i) as suggested earlier, an intensive management of cereal field margins that would override and mask the effect of the landscape context, (ii) a confounding effect of margin width and landscape complexity (i.e., wider margins were located in the more complex landscapes) that would hinder the detection of landscape-scale effects (Figure 2), and/or (iii) the general low representation of alternative habitats (Table 1) enlarging the plant species pool, so that local plant species pools may not have differed largely along our landscape gradient.

Finally, our expectation was that the width and landscape context of margins would act as “environmental filters” of functional traits in margin plant assemblages. Previous studies have provided evidence that agricultural intensification could select for arable plants both within the field [15,28] and in field margins [16,30]. However, in our study, the representation of individual functional trait values was unaffected by margin width or landscape features. There are two plausible explanations for this lack of response. First, as mentioned earlier, most field margins under focus here were strongly affected by disturbances, as suggested by the dominance of therophytes and short-lived perennial species (Table 2). This disturbance regime could have impeded the establishment of long-lived perennial plants, including woody species [42]. It is, therefore, not surprising that the response most commonly reported in the literature, i.e., an increase in perennial and woody species in wide margins and in a complex landscape [15,16,28] could not be detected in the present study. Second, most of the margins studied were located within simple landscapes, and the gradient of landscape complexity under study here may have been too short to filter out functional trait values.

## 4. Materials and Methods

### 4.1. Study Area

**Figure 3:**
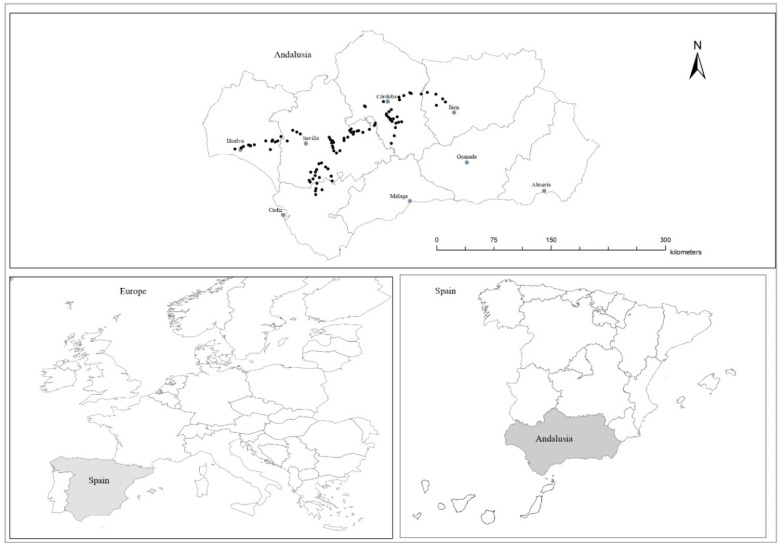
Map showing the location of the 94 sampled margins.

The study area was located in the Guadalquivir river basin (Andalusia, Southern Spain; Figure 3). Land use is dominated by cereal crops, followed by olive orchards and other annual crops such as sunflower or cotton [43]. Natural habitats, such as Mediterranean woodland and scrubland, represent a minor land use and are composed of small unconnected patches [22,43]. The climate in the study area is Mediterranean, with an average annual temperature of 18.6 °C and an average annual precipitation of 590 mm. Altitude ranges from 12 to 106 m a.s.l. Soils devoted to cereal cropping are alkaline with a texture varying from clayish to sandy loam.

### 4.2. Field Margin Selection

Ninety-four field margins adjacent to conventionally managed winter cereal fields were selected. Selected margins were located at least 2 km away from each other in order to avoid overlapping landscape buffers.

### 4.3. Margin Width and Landscape Features as Measures of Agricultural Intensification

Margin width was considered as an indicator of the intensity of agricultural management at the local scale and was measured in-situ during the plant surveys. Margin width ranged from a few centimetres to more than three meters. Depending on width, the margins were categorized as narrow, medium-sized or wide margins (Table 5).

Six variables, used as proxies of the intensity of agricultural practices at the landscape level, were assessed within 1 km radius buffers around each margin [44] using the Geographic Information System SIGPAC (Sistema de Información Geográfica de Parcelas Agrícolas; http://sigpac.mapa.es/fega/visor/): (i) arable land cover, (ii) annual grassland cover, (iii) woodland cover, (iv) cover of human settlements (Table 5), (v) the Shannon–Wiener’s diversity index for land use, and (vi) the size of the adjacent field. Cover of perennial crops (olive orchards and fruit tree crops) was strongly correlated with arable land cover (r = −0.87, *p* < 0.0001) and was thus not included as an additional explanatory variable.

### 4.4. Plant Surveys

Plant surveys were conducted during the ripening stage of winter cereals, between mid-April and early June, in 2009, 2010 and 2011. In each margin, plant species were recorded in an area of 20 m × margin width. The number of species recorded was used as a measure of species richness. Plant nomenclature followed [33].

### 4.5. Plant Functional Traits

A set of five functional traits related to plant persistence, resource acquisition and reproduction was selected:

(i) Raunkiær’s life form: therophytes, geophytes and hemicryptophytes. This trait signals the strategy for plant persistence [42,45]; (ii) growth form: forbs and grasses. This trait is related to plant architecture, resource acquisition and tolerance to selective herbicides [44,46]; (iii) pollination type: entomogamy, anemogamy and autogamy; (iv) seed dispersal mode: zoochory, anemochory and barochory. Both pollination type and seed dispersal mode categories represent contrasting strategies for pollen and seed dispersal in space and time, and are related to colonization ability [32,47,48]; and (v) seed mass, a trait related to reproductive investment, seedling establishment ability and persistence in the soil seed bank [49,50].

Specific values for each trait were retrieved from existing databases (Table 1 and Appendix B). To avoid the influence of rare species, only species recorded in at least 10% of the margins were considered in subsequent analyses [51,52]. The average seed mass of cogeneric species was used for three species for which no seed mass data were available.

### 4.6. Functional Diversity

The Rao’s quadratic entropy index [53] was used to measure plant functional diversity (FD) in field margins [54]. This index incorporates both the relative abundance of species and a measure of the pair-wise functional differences between species, by measuring species distance in the functional trait space:

(1) $$\mathrm{Rao}=\overset{s}{\underset{i=1}{\sum }}\overset{s}{\underset{j=1}{\sum }}d_{ij}p_{i}p_{j}$$

where *s* is the number of species, *d_ij_* is the distance in trait values between species *i* and *j*, and *p_i_* and *p_j_* are the relative abundances of species *i* and *j*. We used species presence/absence as our abundance measure, with present and absent species assigned an abundance value of 1 and 0, respectively. In this way, Rao’s index is largely a measure of functional richness, i.e., the volume of niche space occupied by the species [55]. Rao’s index was calculated for each single trait and for the full trait set (FDT).

### 4.7. Functional Diversity

A principal component analysis (PCA) [56], including the local and landscape variables (Table 1), was conducted after standardization (centering and scaling) in an attempt to produce synthetic variables representing the gradient of agricultural intensification across the 94 sampled margins. The association of each intensification variable to selected PCA axes was measured by the Spearman’s rank-order correlation coefficient (quantitative variables) or the Kruskal–Wallis test (margin types according to width).

The influence of margin width type on species richness and functional diversity measures was tested by the Kruskal–Wallis test. The effect of each individual landscape variable and selected PCA axes on species richness and functional diversity was assessed by Spearman’s rank correlation coefficient.

Two complementary three-table analyses, RLQ and fourth-corner, were further conducted to associate plant traits with local and landscape variables measuring the intensity of agricultural practices [57] (Appendix C). RLQ analysis is a multivariate technique that provides trait combinations that have the highest covariance with combinations of environmental variables [58]. Fourth-corner analysis tests relationships between individual functional traits and individual environmental variables [59]. Both analyses are thus complementary and require three tables, the environment table (R, the local and landscape variables measured on the 94 sampled margins), the species composition table (L, the species present in the sampled margins at ≥ 10% frequency) and the trait-species table (Q, the species values for the selected functional traits, Appendix B). The RLQ analysis performs a simultaneous ordination of the three tables in different steps. First, correspondence analysis (CA) and Hill and Smith analyses were used to analyze respectively the L, R (with row weights equal to the row weights of CA), and the Q (with row weights equal to the column weights of CA) tables. RLQ then calculated two separate co-inertias on the R-L and L-Q tables and identified axes in which the species scores were rearranged to maximize the covariance between the sampling units constrained by the explanatory variables (the R table), and the species scores constrained by the species traits (the Q table); this resulted in linear combinations of functional traits and the explanatory variables. A permutation model (model 6 with 999 permutations, as proposed by [60]) with Bonferroni correction for multiple comparisons was used to test the link between species traits and the environment. This permutation model encompasses two sub-models, models 2 and 4, which test the hypotheses that species presence is independent from their environment (row permutation of the R table) and from their traits (row permutation of the Q table), respectively. Both sub-models must be rejected to confirm the relationship between R and Q tables. The fourth-corner analysis then assessed the association between a pair of quantitative variables with the Pearson correlation coefficient, between a pair of qualitative variables with the Pearson chi-square and G statistic, and between one quantitative-one qualitative variable with the Pseudo-F and Pearson correlation coefficient. The significance of these relationships was tested by 999 permutations based on model 6 with Bonferroni correction for multiple testing.

All statistical analyses were performed with R (version 2.15.1) [61], using the libraries Ade4 [62], Hmisc [63] and pgirmess [64].

## 5. Conclusions

Even though a total of 306 species were recorded in this study, it appears that the structure and management of the studied margins are currently the main factors limiting their plant taxonomic and functional diversity. Despite the selection of margins located along gradients of margin width and landscape complexity, plant assemblages of the selected margins were functionally not diversified, with a clear dominance of therophytes (of which many were pernicious weeds) and limited occurrence of perennial species. Such low functional diversity is most likely a result of the inability of these highly simplified habitats for buffering the effects of intensive field management practices. Promoting species richness and functional diversity of dryland cereal field margins in the study area could, therefore, be achieved by widening existing margins so that some woody species can establish and the proliferation of weed species can be limited. Further investigations are needed to establish the importance of margin width as a management tool aimed to conserve plant diversity in rain-fed cereal field margins.

## Figures and Tables

**Table 1:** List of qualitative and quantitative traits used for the 58 species analyzed.

Traits	Category	Abbreviation	Number of Species	Min.	Max.	Mean ± SD	Source
Raunkiær’s life forms	Geophytes	geop	1	-	-	-	[33]
	Hemicryptophytes	hemi	10	-	-	-	[33]
	Therophytes	thero	47	-	-	-	[33]
Growth form	Forbs	forb	44	-	-	-	[33]
	Grasses	gras	14	-	-	-	[33]
Pollination	Anemogamy	aneg	18	-	-	-	[34]
	Autogamy	auto	3	-	-	-	[34]
	Entomogamy	ento	37	-	-	-	[34]
Seed dispersal type	Anemochory	anem	18	-	-	-	[33,35]
	Barochory	baro	28	-	-	-	[33,35]
	Zoochory	zooc	12	-	-	-	[33,35]
Seed mass (mg)	-	sm	58	0.05	22.5	3.70 ± 5.20	[36]

SD = standard deviation.

**Table 2:** Correlations of PCA first axis (PC1) with the landscape and local variables selected as proxies of the intensity of management in field margins. Spearman’s rank correlations (ρ) were used except for margin width class, for which the correlation with PC1 was determined by the Kruskal–Wallis (χ^2^) test.

	ρ	χ^2^	*p*-Value
Arable land	0.84		<0.001
Field Size	0.28		<0.004
Shannon index for land use diversity	−0.58		<0.001
Woodland cover	−0.33		0.001
Grassland cover	−0.27		0.007
Cover of human settlements	−0.58		0.002
Margin width class		F = 2.03	<0.001

**Table 3:** Correlations of species’ richness, functional diversity for the whole trait set (FDT) and for individual traits (FDS) of field margin plant assemblages with PCA first axis (PC1) and with landscape and local variables. Spearman’s rank correlations (ρ) were used except for margin width class, for which correlations were determined by the Kruskal-Wallis (χ^2^) test. In bold, *p*-values < 0.05.

	PCA1	Arable Land Cover	Field Size	Shannon Index for Land Use Diversity	Woodland Cover	Grassland Cover	Human Settlements Cover	Margin Width Class
Species richness	ρ = 0.06	ρ = 0.09	ρ = 0.17	ρ = −0.11	ρ = −0.04	ρ = 0.09	ρ = 0.05	**χ^2^ = 31.70**
FDT	ρ = 0.03	ρ = 0.08	ρ = −0.07	ρ = −0.10	ρ = 0.00	ρ = −0.03	ρ = 0.09	**χ^2^ = 6.45**
FDS-Raunkiær’s life form	ρ = 0.07	ρ = 0.01	ρ = −0.02	ρ = 0.02	ρ = 0.00	ρ = −0.01	ρ = 0.19	χ^2^ = 2.74
FDS-growth form	ρ = 0.04	ρ = 0.06	ρ = −0.04	ρ = 0.02	ρ = 0.05	ρ = 0.03	ρ = −0.01	χ^2^ = 0.36
FDS-pollination type	ρ = 0.14	ρ = −0.15	ρ = −0.17	ρ = 0.02	ρ = 0.13	ρ = −0.03	ρ = −0.07	χ^2^ = 2.43
FDS-seed dispersal mode	ρ = 0.07	ρ = 0.05	ρ = 0.05	ρ = 0.03	ρ = 0.03	ρ = −0.11	ρ = −0.06	**χ^2^ = 16.50**
FDS-seed mass	ρ = 0.02	ρ = 0.03	ρ = 0.00	ρ = −0.04	ρ = −0.07	ρ = −0.02	ρ = 0.07	χ^2^ = 2.29

**Table 4:** Relationships between functional traits and intensification variables provided by the fourth-corner analysis. None of the relationships were significant (*p* > 0.05 in all cases).

	Arable Land Cover	Grassland Cover	Woodland Cover	Human Settlements Cover	Shannon Index for Land Use Diversity	Field Size	Margin Width Class
Raunkiær’s life forms	F = 0.40	F = 0.32	F = 0.90	F = 0.23	F = 0.00	F = 2.37	χ^2^ = 4.61
Growth form	F = 0.50	F = 0.87	F = 0.46	F = 0.25	F = 2.86	F = 0.13	χ^2^ = 1.61
Pollination	F = 1.26	F = 0.19	F = 0.25	F = 0.25	F = 1.99	F = 0.43	χ^2^ = 3.24
Seed dispersal type	F = 1.26	F = 0.92	F = 1.91	F = 0.55	F = 0.42	F = 0.11	χ^2^ = 6.52
Seed mass	r = −0.01	r = −0.01	r = 0.01	r = 0.00	r = 0.00	r = −0.1	F = 0.55

For two quantitative variables, Pearson correlation coefficient (r) was used. For one quantitative and one qualitative variable pseudo-F was employed (F). For two qualitative variables, chi-square test was used (χ^2^).

**Table 5:** Agricultural intensification variables of the 94 studied field margins.

Landscape Variables	Abbreviation	Category	Min.	Max.	Mean ± SD	Frequency
Arable land cover (%)	AL		2.00	100	73.8 ± 29.90	94
Field Size (ha)	FS		0.16	281.00	9.0 ± 49.10	94
Shannon habitat diversity index	SHDI		0.00	1.10	0.5 ± 0.30	94
Forest (%)	FO		0.00	30.00	0.9 ± 3.70	13
Grassland (%)	GR		0.00	47.00	0.9 ± 5.10	12
Human settlements (%)	HS		0.00	40.00	4.0 ± 6.10	74
Margin width	width	Narrow	0 m	0.99 m	-	37
		Medium	1	1.99 m	-	26
		Wide	>2 m		-	31

SD = standard deviation.

## Data Availability

Data will be provided upon request to the corresponding author.

## Appendix A. List of the 306 Species Found in the 94 Field Margins and Their Frequency. The Species Are Sorted Alphabetically. In Bold, Are the 58 Species Considered in the RLQ, Fourth Corner and in the Functional Diversity Calculations

*Aegilops geniculata* Roth (2%)

*Aegilops triuncialis* L. (1%)

*Ajuga iva* (L.) Schreb. (1%)

*Allium ampeloprasum* L. (1%)

*Amaranthus albus* L. (1%)

*Amaranthus blitoides* S. Watson (9%)

*Amaranthus retroflexus* L. (3%)

*Ammi majus* L. (3%)

***Anacyclus clavatus* (Desf.) Pers. (11%)**

***Anacyclus radiatus* Loisel. (17%)**

***Anagallis arvensis* L. (50%)**

*Anagallis foemina* Mill. (2%)

*Anchusa azurea* Mill. (3%)

***Andryala integrifolia* L. (20%)**

*Anthemis arvensis* L. (4%)

*Anthemis cotula* L. (2%)

*Apera spica venti* (L.) P. Beauv. (1%)

*Apium nodiflorum* (L.) Lag. (1%)

Appiaceae (1%)

*Aristolochia baetica* L. (1%)

*Arundo donax* L. (1%)

*Arum* sp. (1%)

*Asparagus acutifolius* L. (1%)

*Asparagus officinalis* L. (2%)

*Astragalus hamosus* L. (4%)

*Atractylis cancellata* L. (1%)

*Atriplex prostrata* L. (3%)

*Avena barbata* Link (5%)

***Avena sterilis* L. (56%)**

*Bartsia trixago* L. (2%)

***Beta vulgaris* L. (15%)**

*Biscutella auriculata* L. (1%)

*Borago officinalis* L. (8%)

*Brachypodium phoenicoides* (L.) Roem. & Schult. (1%)

***Bromus diandrus* Roth (26%)**

***Bromus hordeaceus* L. (23%)**

***Bromus lanceolatus* Roth (11%)**

***Bromus madritensis* L. (53%)**

*Bromus rubens* L. (4%)

*Bromus tectorum* L. (1%)

*Bromus* sp. (1%)

*Buglossoides arvensis* (L.) I. M. Johnston (2%)

***Calendula arvensis* L. (10%)**

***Campanula erinus* L. (12%)**

*Campanula lusitanica* L. (1%)

*Capnophyllum peregrinum* (L.) Lag. (2%)

*Capsella bursa pastoris* (L.) Medik. (6%)

*Cardaria draba* (L.) Desv. (1%)

*Carduncellus caeruleus* (L.) C. Presl (1%)

*Carduus burgeanus* Boiss. & Reut. (7%)

*Carduus pycnocephalus* L. (6%)

*Carduus tenuiflorus* Curtis (2%)

*Carduus* sp. (3%)

*Carthamus lanatus* L. (3%)

*Catapodium rigidum* (L.) C. E. Hubb. (8%)

*Centaurea calcitrapa* L. (1%)

*Centaurea diluta Aiton* (9%)

***Centaurea melitensis* L. (10%)**

*Centaurea pullata* L. (2%)

*Centaurium erythraea* Rafn, Danm, Holst (1%)

*Centaurium pulchellum* (Sw.) Druce (2%)

*Cerastium glomeratum* Thuill. (2%)

*Chamaemelum fuscatum* (Brot.) Vasc. (3%)

*Chamaemelum nobile* (L.) All. (2%)

*Chamaesyce nutans* (Lag.) Smal (1%)

*Chamaesyce prostrate* (Aiton) Small (1%)

*Chenopodium album* L. (8%)

*Chenopodium opulifolium* Koch & Ziz (1%)

*Chenopodium* sp. (1%)

***Chenopodium vulvaria* L. (12%)**

***Chrozophora tinctoria* (L.) Raf. (15%)**

***Cinchorium intybus* L. (21%)**

*Cladanthus mixtus* (L.) Chevall. (4%)

*Conium maculatum* L. (1%)

*Convolvulus altheoides* L. (4%)

***Convolvulus arvensis* L. (43%)**

*Conyza bonariensis* (L.) Cronq. (9%)

*Conyza canadensis* (L.) Cronq. (6%)

*Conyza sumatrensis* (Retz.) E. Walker (8%)

*Conyza* sp. (1%)

*Coronilla scorpioides* (L.) W. D. J. Koch (3%)

*Crepis capillaris* (L.) Wallr. (1%)

*Crepis foetida* L. (1%)

***Crepis vesicaria* L. (11%)**

*Crypsis* sp. (1%)

*Cuscuta campestris* Yunck. (1%)

*Cynara cardunculus* L. (1%)

***Cynodon dactylon* (L.) Pers. (49%)**

*Cynoglossum creticum* Mill. (1%)

*Cyperus rotundus* L. (2%)

*Dactylis glomerata* L. (3%)

*Datura stramonium* L. (1%)

***Daucus carota* L. (19%)**

*Desmazeria rigida* (4%)

***Diplotaxis virgata* (Cav.) DC. (45%)**

***Ecballium elaterium* (L.) A. Rich. (16%)**

*Echinochloa colonum* (L.) Link. (1%)

*Echinops strigosus* L. (2%)

*Echium arenarium* Guss. (1%)

*Echium creticum* L. (1%)

***Echium plantagineum* L. (27%)**

*Echium* sp. (1%)

*Elymus repens* (L.) Gould (3%)

*Emex spinosa* (L.) Campd. (1%)

*Erodium cicutarium* (L.) L’Hér. (8%)

***Erodium malacoides* (L.) L’Hér. (12%)**

***Erodium moschatum* (L.) L’Hér. (16%)**

*Eryngium campestre* L. (6%)

***Euphorbia exigua* L. (10%)**

*Euphorbia helioscopia* L. (6%)

*Euphorbia nutans* (1%)

*Euphorbia prostrata* L. (1%)

*Fallopia convolvulus* (L.) Á. Löve (3%)

*Fedia scorpioides* Dufresne (1%)

*Filago pyramidata* L. (7%)

*Foeniculum vulgare* Mill. (3%)

*Frankenia laevis* L. Ch (1%)

*Fumaria agraria* Lag. (3%)

*Fumaria faurei* (Pugsley) Lidén (1%)

*Fumaria officinalis* L. (4%)

*Fumaria parviflora* Lam. (3%)

*Fumaria* sp. (2%)

*Galactites tomentosa* Moench (6%)

***Galium aparine* L. (27%)**

*Galium divaricatum* Pourret ex Lam. (4%)

*Galium murale* (L.) All. (2%)

***Galium parisiense* L. (10%)**

*Galium spurium* L. (4%)

*Galium tricornutum* Dandy (9%)

*Galium verrucosum* Huds. (3%)

*Galium* sp. (1%)

*Gaudinia fragilis* (L.) P. Beauv (1%)

*Geranium disectum* L. (4%)

*Geranium molle* L. (2%)

*Glaucium corniculatum* (L.) Rudolph (1%)

***Glebionis coronaria* (L.) Spach (47%)**

***Glebionis segetum* (L.) Fourr. (13%)**

*Hainardia cylindrica* (Willd.) Greuter (3%)

*Hedypnois cretica* (L.) Dum.–Cours. (3%)

***Heliotropium europaeum* L. (13%)**

***Helminthotheca echioides* (L.) (38%)**

*Herniaria cinerea* DC. (8%)

***Hirschfeldia incana* (L.) Lagr. Foss. (15%)**

*Holcus lanatus* L. (1%)

*Hordeum leporinum* Link (1%)

*Hordeum marinum* Huds. (1%)

*Hordeum* sp. (1%)

***Hordeum murinum* L. (51%)**

*Juncus bufonius* L. (6%)

*Kickxia spuria* (L.) Dumort. (2%)

*Kickia* sp. (2%)

***Lactuca serriola* L. (30%)**

*Lamarckia aurea* (L.) Moench (5%)

*Lamium amplexicaule* L. (4%)

*Lathyrus cicera* L. (1%)

*Lathyrus hirsutus* L. (1%)

***Lavatera cretica* L. (34%)**

*Lavatera* sp. (2%)

*Lavatera trimestris* L. (6%)

*Leontodon longirrostris* (Finch & P. D. Sell) Talavera (1%)

*Leontodon maroccanus* (Pers.) Ball (1%)

*Linaria latifolia* Desf. (4%)

*Linaria spartea* (L.) Chaz. (1%)

*Linum* sp. (1%)

*Linum tenue* Desf. (1%)

*Lolium multiflorum* Lam (1%)

***Lolium rigidum* Gaudin (73%)**

*Lotus subbiflorus* Lag. (1%)

*Lupinus angustifolius* L. (2%)

*Lythrum acutangulum* Lag. (5%)

*Lythrum hyssopifolia* L. (2%)

*Lythrum junceum* Banks & Sol. (6%)

*Malva hispanica* L. (1%)

*Malva intermedia* Boreau (1%)

***Malva nicaensis* All. (25%)**

***Malva parviflora* L. (36%)**

***Malva sylvestris* L. (17%)**

*Malva* sp. (9%)

*Malvella sherardiana* (L.) Jaub. & Spach (1%)

*Marrubium vulgare* L. (1%)

*Medicago ciliaris* (L.) All. (1%)

*Medicago orbicularis* (L.) Bartal. (1%)

***Medicago polymorpha* L. (17%)**

*Medicago sativa* L. (1%)

*Medicago scutellata* (L.) Mill. (1%)

*Medicago* sp. (4%)

*Melilotus indicus* (L.) All. (6%)

*Mentha suaveolens* Ehrh. (1%)

*Mercurialis ambigua* L. (1%)

*Misopates orontium* (L.) Raf. (7%)

Misopates cretica (1%)

*Misopates* sp. (1%)

*Nigella papillosa* G. López (3%)

*Notobasis syriaca* (L.) Cass. (1%)

*Ononis mitissima* L. (7%)

*Ononis natrix* L. Gh. (1%)

*Ononis* sp. (1%)

*Onopordum nervosum* Boiss. (1%)

*Ornithogalum narbonense* L. (2%)

*Ornithopus compresus* L. (1%)

*Orobanche ramosa* L. (1%)

*Osyris alba* L. Np. (1%)

*Pallenis spinosa* (L.) Cass. (3%)

*Papaver dubium* L. (1%)

*Papaver hybridum* L. (4%)

*Papaver pinnatifidum* Moris (1%)

***Papaver rhoeas* L. (24%)**

*Parapholis incurva* (L.) C. E. Hubb. (1%)

*Parapholis pycnantha* (Druce) C. E. Hubb. (1%)

***Phalaris brachystachys* Link (24%)**

*Phalaris coerulescens* Desf. (3%)

***Phalaris minor* Retz (47%)**

***Phalaris paradoxa* L. (45%)**

*Phalaris* sp. (1%)

***Piptatherum miliaceum* (L.) Coss. (16%)**

*Plantago afra* L. (7%)

*Plantago albicans* L. (1%)

*Plantago coronopus* L. (2%)

***Plantago lagopus* L. (17%)**

*Plantago lanceolata* L. (4%)

*Podospermum lancinatum* L. (1%)

*Polycarpon tetraphyllum* (L.) L. (6%)

***Polygonum aviculare* L. (41%)**

*Polygonum bellardii* All. (1%)

*Polypogon maritimus* Willd. (1%)

***Polypogon monspeliensis* (L.) Desf. (27%)**

*Portulaca oleracea* L. (4%)

***Pulicaria paludosa* Link (39%)**

*Ranunculus arvensis* L. (1%)

*Raphanus raphanistrum* L. (6%)

***Rapistrum rugosum* (L.) All. (10%)**

*Reseda luteola* L. (6%)

***Ridolfia segetum* (L.) Moris (20%)**

*Rostraria cristata* (L.) Tzvelev (8%)

*Rumex conglomeratus* Murray (1%)

*Rumex crispus* L. (3%)

*Rumex obtusifolius* L. (1%)

*Rumex pulcher* L. (8%)

*Sagina apetala* Ard. (1%)

*Scabiosa atropurpurea* L. (1%)

*Scolymus hispanicus* L. (5%)

***Scolymus maculatus* L. (20%)**

*Scorpiurus muricatus* L. (1%)

*Scorpiurus sulcatus* L. (4%)

*Scorpiurus vermiculatus* L. (2%)

*Scorzonera laciniata* L. (1%)

*Setaria* sp. (2%)

*Setaria viridis* (L.) P. Beauv. (1%)

*Sherardia arvensis* L. (5%)

*Silene gallica* L. (3%)

*Silene nocturna* L. (1%)

*Silene stricta* L. (2%)

***Silybum marianum* (L.) Gaertn. (29%)**

*Sinapis alba* L. (6%)

*Sinapis arvensis* L. (3%)

*Sisymbrium officinale* (L.) Scop. (1%)

*Solanum nigrum* L. (2%)

*Sonchus asper* (L.) Hill (5%)

***Sonchus oleraceous* L. (48%)**

*Sorghum halepense* (L.) Pers. (1%)

*Spergula arvensis* L. (1%)

*Spergularia bocconei* (Scheele) Graebn. (1%)

*Spergularia nicaeensis* Burnat (3%)

*Spergularia rubra* (L.) J. Presl & C. Presl (2%)

*Spergularia* sp. (1%)

*Stachys arvensis* (L.) L. (3%)

*Stachys ocymastrum* (L.) Briq. (2%)

*Symphyotrichum squamatum* (Spreng.) G. L. Nesom (5%)

*Taraxacum officinale* Weber (2%)

*Teucrium capitatum* L. Ch. (1%)

*Tolpis barbata* (L.) Gaertn. (1%)

***Torilis arvensis* (Huds.) Link (14%)**

*Torilis nodosa* (L.) Gaertn. (9%)

*Torilis* sp. (1%)

*Trachynia distachya* (L.) Link (6%)

*Tragopogon crocifolius* L. (1%)

*Trifolim angustifolim* L. (2%)

*Trifolium campestre* Schreb. (5%)

*Trifolium glomeratum* L. (2%)

*Trifolium repens* L. (2%)

*Trifolium resupinatum* L. (1%)

*Trifolium scabrum* L. (1%)

*Trifolium* sp. (2%)

*Trifolium squamosum* L. (1%)

*Trifolium tomentosum* L. (1%)

*Trifolium vesiculosum* Savi (3%)

*Trifolium* sp. (1%)

***Trisetaria panicea* (Lam.) Paunero (30%)**

***Urospermun picrioides* (L.) F. W. Schmidt (12%)**

*Urtica urens* L. (1%)

*Vaccaria hispanica* (Mill.) Rauschert (1%)

*Verbascum sinuatum* L. (3%)

*Verbena officinalis* L. (1%)

*Verbena supina* L. (1%)

*Veronica anagalloides* Guss. (1%)

*Veronica arvensis* L. (2%)

*Veronica polita* Fr. (4%)

*Vicia lutea* L. (1%)

*Vicia sativa* L. (4%)

*Vicia* sp. (1%)

*Vulpia ciliata* Dumort. (1%)

*Vulpia geniculata* (L.) Link (9%)

*Vulpia* sp. (2%)

*Vulpia myuros* (L.) C. C. Gmel. (3%)

*Xanthium spinosum* L. (1%)

*Xanthium strumarium* L. (4%)

## Appendix B. Species Traits (Q Table)

**Species**	**Life Form**	**Growth Form**	**Pollination**	**Seed Dispersal Type**	**Seed Mass (mg)**
Anacyclus clavatus (Desf.) Pers.	therophyte	forb	entomogamy	anemochory	0.50
Anacyclus radiatus Loisel.	therophyte	forb	entomogamy	anemochory	1.07
Anagallis arvensis L.	therophyte	forb	entomogamy	barochory	0.50
Andryala integrifolia L.	hemicryptophyte	forb	entomogamy	anemochory	0.15
Avena sterilis L.	therophyte	grass	anemogamy	zoochory	19.94
Beta vulgaris L.	therophyte	forb	anemogamy	barochory	12.70
Bromus diandrus Roth	therophyte	grass	anemogamy	zoochory	11.24
Bromus lanceolatus Roth	therophyte	grass	anemogamy	zoochory	3.90
Bromus hordeaceus L.	therophyte	grass	anemogamy	zoochory	1.48
Bromus madritensis L.	therophyte	grass	anemogamy	zoochory	3.33
Calendula arvensis L.	therophyte	forb	entomogamy	zoochory	5.20
Campanula erinus L.	therophyte	forb	entomogamy	anemochory	0.09 *
Centaurea melitensis L.	therophyte	forb	entomogamy	anemochory	1.40
Chenopodium vulvaria L.	therophyte	forb	anemogamy	barochory	0.40
Chrozophora tinctoria (L.) Raf.	therophyte	forb	entomogamy	barochory	16.00
Cinchorium intybus L.	hemicryptophyte	forb	entomogamy	anemochory	5.50
Convolvulus arvensis L.	geophyte	forb	entomogamy	barochory	15.10
Crepis vesicaria L.	hemicryptophyte	forb	entomogamy	anemochory	0.36
Cynodon dactylon (L.) Pers.	hemicryptophyte	grass	anemogamy	barochory	0.20
Daucus carota L.	hemicryptophyte	forb	entomogamy	zoochory	1.00
Diplotaxis virgata (Cav.) DC.	therophyte	forb	entomogamy	anemochory	0.23 *
Ecballium elaterium (L.) A. Rich.	hemicryptophyte	forb	entomogamy	barochory	12.10
Echium plantagineum L.	therophyte	forb	entomogamy	barochory	4.30
Erodium malacoides (L.) L’Hér	therophyte	forb	entomogamy	barochory	1.40
Erodium moschatum (L.) L’Hér.	therophyte	forb	entomogamy	barochory	2.62
Euphorbia exigua L.	therophyte	forb	anemogamy	barochory	0.35
Galium aparine L.	therophyte	forb	entomogamy	zoochory	8.70
Galium parisiense L.	therophyte	forb	entomogamy	zoochory	0.20
Glebionis coronaria (L.) Spach	therophyte	forb	entomogamy	anemochory	1.50
Glebionis segetum (L.) Fourr.	therophyte	forb	entomogamy	anemochory	1.52
Heliotropium europaeum L.	therophyte	forb	entomogamy	barochory	1.10
Helminthotheca echioides (L.) Holub	hemicryptophyte	forb	entomogamy	anemochory	1.31
Hirschfeldia incana (L.) Lagr. Foss.	therophyte	forb	entomogamy	barochory	0.23
Hordeum murinum L.	therophyte	grass	anemogamy	zoochory	10.50
Lactuca serriola L.	therophyte	forb	autogamy	anemochory	0.58
Lavatera cretica L.	therophyte	forb	entomogamy	barochory	7.01
Lolium rigidum Gaudin	therophyte	grass	anemogamy	barochory	3.34
Malva nicaensis All.	therophyte	forb	entomogamy	barochory	8.60
Malva parviflora L.	therophyte	forb	entomogamy	barochory	2.80
Malva sylvestris L.	therophyte	forb	entomogamy	barochory	5.40
Medicago polymorpha L.	therophyte	forb	autogamy	zoochory	2.95
Papaver rhoeas L.	therophyte	forb	entomogamy	anemochory	0.20
Phalaris brachystachys Link	therophyte	grass	anemogamy	barochory	1.90
Phalaris minor Retz.	therophyte	grass	anemogamy	barochory	1.60
Phalaris paradoxa L.	therophyte	grass	anemogamy	barochory	1.30
Piptatherum miliaceum (L.) Coss.	hemicryptophyte	grass	anemogamy	barochory	0.61
Plantago lagopus L.	hemicryptophyte	forb	anemogamy	barochory	0.30
Polygonum aviculare L.	therophyte	forb	autogamy	barochory	1.30
Polypogon monspeliensis (L.) Desf.	therophyte	grass	anemogamy	barochory	0.10
Pulicaria paludosa Link	therophyte	forb	entomogamy	anemochory	0.17 *
Rapistrum rugosum (L.) All.	therophyte	forb	entomogamy	barochory	2.90
Ridolfia segetum (L.) Moris	therophyte	forb	entomogamy	barochory	0.60
Scolymus maculatus L.	therophyte	forb	entomogamy	anemochory	1.54
Sonchus oleraceous L	therophyte	forb	entomogamy	anemochory	0.30
Silybum marianum (L.) Gaertn.	hemicryptophyte	forb	entomogamy	anemochory	22.50
Torilis arvensis (Huds.) Link	therophyte	forb	entomogamy	zoochory	2.10
Trisetaria panicea (Lam.) Paunero	therophyte	grass	anemogamy	barochory	0.05
Urospermum picrioides (L.) F. W. Schmidt	Therophyte	forb	entomogamy	anemochory	1.6
* The seed mass value was calculated by computing the average seed mass of congeneric species.					

## Appendix C. R-Scripts to Perform RLQ and Fourth-Corner Analyses

# 1 Upload the 3 tables R (environmental data), L (Species data) and Q (Functional trait data).

# 2 Upload the ade4 library

library (ade4)

# 3 Perform a separate multivariant analysis of each table.

dudiL <- dudi.coa (df = L, scannf = FALSE, nf = 2)

# Note: Correspondence analysis L

dudiR <- dudi.hillsmith (df = R,row.w = dudiL$lw, scannf = FALSE, nf = 2)

# Note: Hill and Smith analysis is similar to PCA but considering categorical variables. row.w = dudiL$lw is to connect to L table.

dudiQ <- dudi.hillsmith (df = Q, row.w = dudiL$cw, scannf = FALSE, nf = 2)

# Note: Hill and smith of Q.

# 4 Perform the RLQ analysis

RLQ <- rlq (dudiR, dudiL, dudiQ, scannf = FALSE, nf = 2)

# 5 RLQ results

summary (RLQ)

randtest (RLQ)

# RLQ figure

plot (RLQ)

# eigenvalues

SUM.eig <- sum (RLQ$eig)

RLQ$eig * 100/SUM.eig # percentage of each axis

# 6 FOURTH CORNER

a <- fourthcorner (R, L, Q, modeltype = 6, nrepet = 999, p.adjust.method.G = “bonferroni”, p.adjust.method.D = “bonferroni”)

summary (a)

Reference: Dray, S., 2013. A Tutorial to Perform Fourth-Corner and RLQ Analyses in R.

## References

[1] Stoate C., Boatman N.D., Borralho R.J., Carvalho C.R., de Snoo G.R., Eden P. (2001). Ecological impacts of arable intensification in Europe. J. Environ. Manag..

[2] Storkey J., Meyer S., Leuschner C., Still K.S. (2012). The impact of agricultural intensification and land use change on the European arable flora. Proc. R. Soc. B Biol. Sci..

[3] Benton T.G., Vickery J.A., Wilson J.D. (2003). Farmland biodiversity: Is habitat heterogeneity the key?. Trends Ecol. Evol..

[4] Tscharntke T., Klein A.M., Kruess A., Steffan-Dewenter I., Thies C. (2005). Landscape perspectives on agricultural intensification and biodiversity–ecosystem service management. Ecol. Lett..

[5] Le Coeur D., Baudry J., Burel F. (1997). Field margins plant assemblages: Variation partitioning between local and landscape factors. Landsc. Urban Plan..

[6] Petit S., Stuart R.C., Gillespie M.K., Barr C.J. (2003). Field boundaries in Great Britain: Stock and change between 1984, 1990 and 1998. J. Environ. Manag..

[7] Baessler C., Klotz S. (2006). Effects of changes in agricultural land-use on landscape structure and arable weed vegetation over the last 50 years. Agric. Ecosyst. Environ..

[8] Ponisio L.C., M’Gonigle L.K., Kremen C. (2016). On-farm habitat restoration counters biotic homogenization in intensively managed agriculture. Glob. Chang. Biol..

[9] Morrison J., Hernández Plaza E., Izquierdo J., Gonzalez-Andujar J.L. (2017). The role of field margins in supporting wild bees in Mediterranean cereal agroecosystems: Which biotic and abiotic factors are important?. Agric. Ecosyst. Environ..

[10] Cirujeda A., Pardo G., Marí A.I., Aibar J., Pallavicini Y., Gonzalez-Andujar J.L., Recasens J., Sole-Senan X.O. (2019). The structural classification of field boundaries in Mediterranean arable cropping systems allows the prediction of weed abundances in the boundary and in the adjacent crop. Weed Res..

[11] Sanchez J.A., Carrasco A., La Spina M., Pérez-Marcos M., Ortiz-Sánchez F.J. (2020). How Bees Respond Differently to Field Margins of Shrubby and Herbaceous Plants in Intensive Agricultural Crops of the Mediterranean Area. Insects.

[12] Marshall E.J.P., Moonen A.C. (2002). Field margins in northern Europe: Their functions and interactions with agriculture. Agric. Ecosyst. Environ..

[13] Cordeau S., Petit S., Reboud X., Chauvel B. (2012). The impact of sown grass strips on the spatial distribution of weed species in adjacent boundaries and arable fields. Agric. Ecosyst. Environ..

[14] Vickery J.A., Feber R.E., Fuller R.J. (2009). Arable field margins managed for biodiversity conservation: A review of food resource provision for farmland birds. Agric. Ecosyst. Environ..

[15] Poggio S.L., Chaneton E.J., Ghersa C.M. (2010). Landscape complexity differentially affects alpha, beta, and gamma diversities of plants occurring in fencerows and crop fields. Biol. Conserv..

[16] Bassa M., Chamorro L., José-María L., Blanco-Moreno J., Sans F. (2012). Factors affecting plant species richness in field boundaries in the Mediterranean region. Biodivers. Conserv..

[17] Schippers P., Joenje W. (2002). Modelling the effect of fertiliser, mowing, disturbance and width on the biodiversity of plant communities of field boundaries. Agric. Ecosyst. Environ..

[18] Tarmi S., Helenius J., Hyvönen T. (2009). Importance of edaphic, spatial and management factors for plant communities of field boundaries. Agric. Ecosyst. Environ..

[19] Marshall E.J.P. (2009). The impact of landscape structure and sown grass margin strips on weed assemblages in arable crops and their boundaries. Weed Res..

[20] José-María L., Armengot L., Blanco-Moreno J.M., Bassa M., Sans F.X. (2010). Effects of agricultural intensification on plant diversity in Mediterranean dryland cereal fields. J. Appl. Ecol..

[21] Jonason D., Andersson G.K.S., Öckinger E., Rundlöf M., Smith H.G., Bengtsson J. (2011). Assessing the effect of the time since transition to organic farming on plants and butterflies. J. Appl. Ecol..

[22] Aparicio A. (2008). Descriptive analysis of the ‘relictual’ Mediterranean landscape in the Guadalquivir River valley (southern Spain): A baseline for scientific research and the development of conservation action plans. Biodivers. Conserv..

[23] Rodríguez C., Wiegand K. (2009). Evaluating the trade-off between machinery efficiency and loss of biodiversity-friendly habitats in arable landscapes: The role of field size. Agric. Ecosyst. Environ..

[24] Flynn D.B., Gogol-Prokurat M., Nogeire T., Molinari N., Trautman Richers B., Lin B.B., Simpson N., Mayfield M.M., DeClerck F. (2009). Loss of functional diversity under land use intensification across multiple taxa. Ecol. Lett..

[25] Keddy P.A. (1992). Assembly and response rules: Two goals for predictive community ecology. J. Veg. Sci..

[26] Diaz S., Cabido M., Casanoves F. (1998). Plant functional traits and environmental filters at a regional scale. J. Veg. Sci..

[27] Díaz S., Cabido M. (2001). Vive la différence: Plant functional diversity matters to ecosystem processes. Trends Ecol. Evol..

[28] José-María L., Blanco-Moreno J.M., Armengot L., Sans F.X. (2011). How does agricultural intensification modulate changes in plant community composition?. Agric. Ecosyst. Environ..

[29] Fried G., Kazakou E., Gaba S. (2012). Trajectories of weed communities explained by traits associated with species’ response to management practices. Agric. Ecosyst. Environ..

[30] Ma M., Herzon I. (2014). Plant functional diversity in agricultural margins and fallow fields varies with landscape complexity level: Conservation implications. J. Nat. Conserv..

[31] Moreno J.C. (2008). Lista Roja 2008 de la Flora Vascular Española.

[32] Benvenuti S. (2007). Weed seed movement and dispersal strategies in the agricultural environment. Weed Biol. Manag..

[33] Blanca G., Cabezudo B., Cueto M., Morales-Torres C., Salazar C. (2011). Flora Vascular de Andalucía Oriental.

[34] Julve P. (1998). Baseflor. Index Botanique Écologique et Chorologique de la Flore de France.

[35] Kleyer M., Bekker R.M., Knevel I.C., Bakker J.P., Thompson K., Sonnenschein M., Poschlod P., Van Groenendael J.M., Klimeš L., Klimešová J. (2008). The LEDA Traitbase: A database of life-history traits of the Northwest European flora. J. Ecol..

[36] Royal Botanic Gardens (2008). Seed Information Database (SID), *Version 7.1*.

[37] Ma M., Tarmi S., Helenius J. (2002). Revisiting the species–area relationship in a semi-natural habitat: Floral richness in agricultural buffer zones in Finland. Agric. Ecosyst. Environ..

[38] Schmitz J., Schäfer K., Brühl C.A. (2014). Agrochemicals in field margins—Field evaluation of plant reproduction effects. Agric. Ecosyst. Environ..

[39] Gonzalez-Andujar J.L., Saavedra M. (2003). Spatial distribution of annual grass weed populations in winter cereals. Crop Prot..

[40] Dainese M., Montecchiari S., Sitzia T., Sigura M., Marini L. (2017). High cover of hedgerows in the landscape supports multiple ecosystem services in Mediterranean cereal fields. J. Appl. Ecol..

[41] Weibull A.C., Östman Ö., Granqvist Å. (2003). Species richness in agroecosystems: The effect of landscape, habitat and farm management. Biodivers. Conserv..

[42] Lososová Z., Chytrý M., Kühn I., Hájek O., Horáková V., Pyšek P., Tichý L. (2006). Patterns of plant traits in annual vegetation of man-made habitats in central Europe. Perspect. Plant Ecol..

[43] de Andalucía J. (2013). Anuario de Estadísticas Agrarias y Pesqueras en Andalucía.

[44] Roschewitz I., Thies C., Tscharntke T. (2005). Are landscape complexity and farm specialisation related to land-use intensity of annual crop fields?. Agric. Ecosyst. Environ..

[45] McIntyre S., Lavorel S., Tremont R.M. (1995). Plant life-history attributes: Their relationship to disturbance response in herbaceous vegetation. J. Ecol..

[46] Hawes C., Squire G.R., Hallett P.D., Watson C.A., Young M. (2010). Arable plant communities as indicators of farming practice. Agric. Ecosyst. Environ..

[47] Holzschuh A., Steffan-Dewenter I., Kleijn D., Tscharntke T. (2007). Diversity of flower-visiting bees in cereal fields: Effects of farming system, landscape composition and regional context. J. Appl. Ecol..

[48] Petit S., Alignier A., Colbach N., Joannon A., Cœur D., Thenail C. (2012). Weed dispersal by farming at various spatial scales. A review. Agron. Sustain. Dev..

[49] Leishman M.R. (2001). Does the seed size/number trade-off model determine plant community structure? An assessment of the model mechanisms and their generality. Oikos.

[50] Pakeman R.J., Garnier E., Lavorel S., Ansquer P., Castro H., Cruz P., Doležal J., Eriksson O., Freitas H., Golodets C. (2008). Impact of abundance weighting on the response of seed traits to climate and land use. J. Ecol..

[51] Mueller-Dombois D., Ellenberg H. (1974). Aims and Methods of Vegetation Ecology.

[52] Kenkel N.C., Derksen D.A., Thomas A.G., Watson P.R. (2002). Review: Multivariate analysis in weed science research. Weed Sci..

[53] Rao C.R. (1982). Diversity and dissimilarity coefficients: A unified approach. Theor. Popul. Biol..

[54] Mouchet M.A., Villéger S., Mason N.W.H., Mouillot D. (2010). Functional diversity measures: An overview of their redundancy and their ability to discriminate community assembly rules. Funct. Ecol..

[55] Mason N.W., Bello F., Mouillot D., Pavoine S., Dray S. (2013). A guide for using functional diversity indices to reveal changes in assembly processes along ecological gradients. J. Veg. Sci..

[56] Hill M.O., Smith A.J.E. (1976). Principal Component Analysis of Taxonomic Data with Multi-State Discrete Characters. Taxon.

[57] Dray S., Choler P., Doledec S., Peres-Neto P.R., Thuiller W., Pavoine S., ter Braak C.J. (2014). Combining the Fourth-corner and the RLQ methods for assessing trait responses to environmental variation. Ecology.

[58] Dolédec S., Chessel D., Braak C.J.F., Champely S. (1996). Matching species traits to environmental variables: A new three-table ordination method. Environ. Ecol. Stat..

[59] Legendre P., Galzin R., Harmelin-Vivien M.L. (1997). Relating behavior to habitat: Solutions to the Fourth-corner problem. Ecology.

[60] Dray S., Legendre P. (2008). Testing the species traits-environment relationships: The fourth-corner problem revisited. Ecology.

[61] RStudio Team (2016). RStudio: Integrated Development for R.

[62] Dray S., Dufour A.B. (2007). The ade4 package: Implementing the duality diagram for ecologists. J. Stat. Softw..

[63] Harrell F., Dupont C. (2014). Hmisc: Harrell Miscellaneous. R Package Version 3.14-0. http://CRAN.R-project.org/package=Hmisc.

[64] Giradoux P. (2013). Pgirmess: Data Analysis in Ecology. R. Package Version 1.5.8. http://CRAN.R-project.org/package=pgirmess.

